# Synthesis of New Cytotoxic Aminoanthraquinone Derivatives *via* Nucleophilic Substitution Reactions

**DOI:** 10.3390/molecules18078046

**Published:** 2013-07-08

**Authors:** Siti Mariam Mohd Nor, Mohd Aspollah Hj Md Sukari, Saripah Salbiah Syed Abdul Azziz, Wong Chee Fah, Hasimah Alimon, Siti Fadilah Juhan

**Affiliations:** 1 Department of Chemistry, Faculty of Science, Universiti Putra Malaysia, 43400 UPM Serdang, Selangor, Malaysia; 2 Department of Chemistry, Faculty of Science and Mathematics, Universiti Pendidikan Sultan Idris, 35900 Tanjong Malim, Perak, Malaysia; 3 Department of Biology, Faculty of Science and Mathematics, Universiti Pendidikan Sultan Idris, 35900 Tanjong Malim, Perak, Malaysia

**Keywords:** methylation, reduction, acylation, amination, substitution, aminoanthraquinone, mechanism, cytotoxic, MCF-7, Hep-G2

## Abstract

Aminoanthraquinones were successfully synthesized *via* two reaction steps. 1,4-Dihydroxyanthraquinone (**1**) was first subjected to methylation, reduction and acylation to give an excellent yield of anthracene-1,4-dione (**3**), 1,4-dimethoxyanthracene-9,10-dione (**5**) and 9,10-dioxo-9,10-dihydroanthracene-1,4-diyl diacetate (**7**). Treatment of **1**, **3**, **5** and **7** with BuNH_2_ in the presence of PhI(OAc)_2_ as catalyst produced seven aminoanthraquinone derivatives **1a**, **b**, **3a**, and **5a**–**d**. Amination of **3** and **5** afforded three new aminoanthraquinones, namely 2-(butylamino)anthracene-1,4-dione (**3a**), 2-(butylamino)anthracene-9,10-dione (**5a**) and 2,3-(dibutylamino)anthracene-9,10-dione (**5b**). All newly synthesised aminoanthraquinones were examined for their cytotoxic activity against MCF-7 (estrogen receptor positive human breast) and Hep-G2 (human hepatocellular liver carcinoma) cancer cells using MTT assay. Aminoanthraquinones **3a**, **5a** and **5b** exhibited strong cytotoxicity towards both cancer cell lines (IC_50_ 1.1–13.0 µg/mL).

## 1. Introduction

Natural and synthetic anthraquinones have attracted the interest of researchers due to their significant biological activities such as antitumour [1,2,3,4], anti-inflammatory [5], antimalarial [5,6,7], antimicrobial [5,8], antifungal [9], antileukemic [10,11], antiviral and anti-HIV properties [12,13,14,15]. Anthraquinone and its derivatives are also used as antioxidants [16], dyes [17,18,19,20] or in photoimaging [20]. 

It has also been reported that amino-substituted anthraquinones show significantly increased antiproliferative activities against human/mammalian cancer cell lines [21,22] and are known to have potential antitumor activity, but are less toxic to normal cells and display low cardiotoxicity [23,24,25,26]. A study on 4-(*N*-cyclohexylamino)-emodin implied that it can discriminate well between hepatoma cells and primary hepatocytes and it retained the capacity to reverse the multi-drug-resistance phenotype [27]. Other aminoanthraquinone derivatives such as Reactive Blue 2 (RB-2), Acid Blue 25 (AB-25) and Acid Blue 129 (AB-129) also known as a good nucleotide-binding proteins [28] where RB-2 is one of the most widely used P2-receptor antagonists [29]. The potential of RB-2 was driven by its hydrophobic interactions of aromatic π-electron systems, and the hydrogen bonds with nitrogen as donor and acceptor atoms as these properties could block the P2-recptor.

Based on these promising bioactivities, the aim of this research was therefore to synthesise some new aminoantharaquinone derivatives with high potential as anticancer and antimicrobial agents. The effects of substrate amount, catalyst amount, reaction time and reaction temperature were studied. According to a study, shorter amines would result in lower cytotoxic effects [24] whereas the use of diamines or longer amine chains allow the possibility of forming side products due to their reactive properties [30]. Therefore, a simple straight chain amine containing four carbons (BuNH_2_) was chosen for this study, while diacetoiodobenzene [PhI(OAc)_2_] was used as catalyst because of it selectivity towards the substitution reaction [24]. Synthesised aminoanthraquinones were then further tested for cytotoxicity and antimicrobial activity. 

## 2. Results and Discussion

The synthesis of aminoanthraquinone derivatives was achieved through two simple reaction steps. 1,4-Dihydroxyanthraquinone (**1**) was first subjected to methylation, reduction, and acylation to produce anthracene-1,4-dione (**3**) 1,4-dimethoxyanthracene-9,10-dione (**5**) and 9,10-dioxo-9,10-dihydro-anthracene-1,4-diyl diacetate (**7**). Compounds **1**, **3**, **5** and **7** were then treated with butylamine (BuNH_2_) using iodobenzenediacetate [PhI(OAc)_2_] as catalyst to produce the desired aminoanthraquinones. All the compounds were elucidated using mp, IR, MS, 1D NMR, 2D NMR and comparison with data in the literature. The proposed mechanisms for the amination are also presented. 

### 2.1. Reduction, Methylation and Acylation

**Scheme 1:**
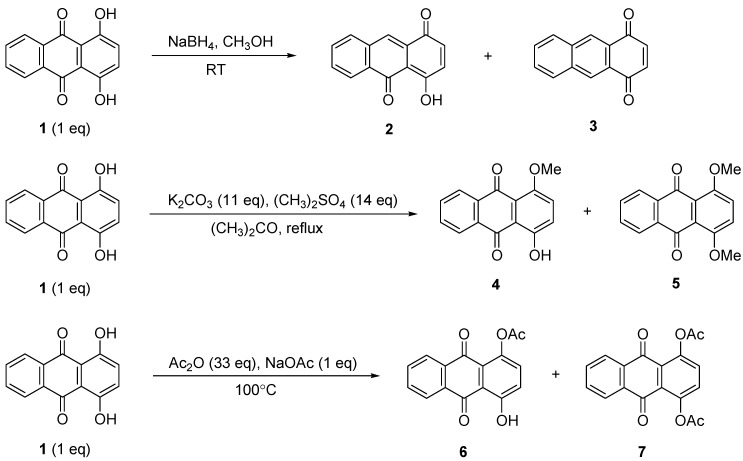
Reduction, methylation and acylation of **1**.

The reduction of compound **1** using NaBH_4_ (1:1 equiv.) was performed successfully in 30 min to give a mixture of 4-hydroxyanthracene-1,10-dione (**2**) and anthracene-1,4-dione (**3**) in 69% and 21% yields, respectively. Increasing the amount of NaBH_4_ to 3 equiv. led to the formation of only compound **3** in excellent yield (Scheme 1 and Table 1). Further NaBH_4_ increases resulted in a lower yield of **3** as the single product obtained (Table 1, Entry I, II and III). 

The methylation of compound **1** with (CH_3_)_2_SO_4_ in the presence of K_2_CO_3_ was achieved in acetone under reflux at 60 °C [31,32]. The used of (CH_3_)_2_SO_4_ as methylating agent is proven to give higher yields [33]. A mixture of 1-hydroxy-4-methoxyanthracene-9,10-dione (**4**) and 1,4-dimethoxy-anthracene-9,10-dione (**5**) were obtained as yellow and orange solids, respectively (Table 1, Entry IV and V). The ^1^H-NMR spectra of compounds **4** and **5** showed singlets at *δ* 3.76 and *δ* 3.94 ppm, respectively, which were attributed to the methoxy protons.

**Table 1:** Some effects on the reduction, methylation and acylation of **1**.

Entry	NaBH_4_ (equiv.)	Reaction Time	Product (% Yield)
I	1	30 min	**2** (69), **3** (21)
II	3	30 min	**3** (90)
III	15	30 min	**3** (60)
IV	-	3 h	**4** (5), **5** (85)
V	-	4 h	**5** (96)
VI	-	2 h	**6** (26), **7** (60)
VII	-	3 h	**6** (15), **7** (75)
VII	-	9 h	**7** (90)

The acylation of compound **1** by using excess acetic acid anhydride in the presence of sodium acetate was completed in 2 h to obtain a mixture of 4-hydroxy-9,10-dioxo-9,10-dihydroanthracen-1-yl acetate (**6**) and 9,10-dioxo-9,10-dihydroanthracene-1,4-diyl diacetate (**7**) (Scheme 1). Increasing the reaction time for both methylation and acylation seemed to increase the dimethylated and diacetylated products, **5** and **7** (Table 1, Entry V, VI, VII and VIII). It was believed that the product of **3**, **5** and **7** were produced through the intermediate compounds **2**, **4** and **6**, respectively.

### 2.2. Amination

**Scheme 2:**
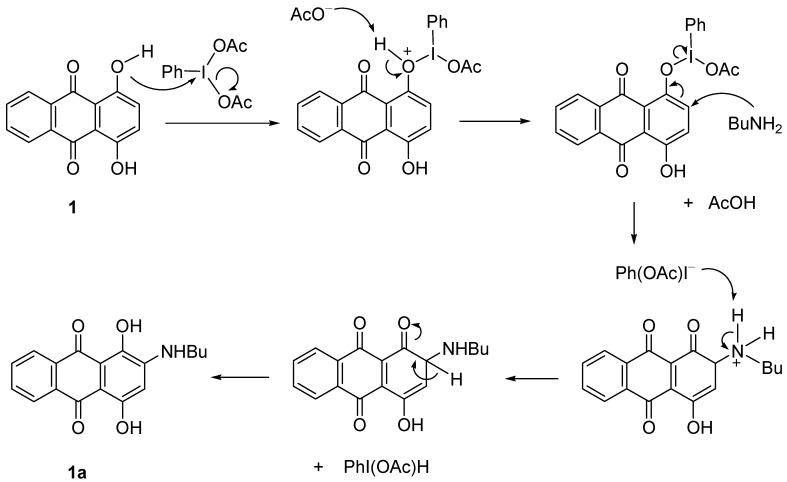
Proposed mechanism of formation of **1a** in the presence of catalyst.

Amination reactions were attempted using procedure of Teich *et al.* [24]. It was stated that the formation of aminoanthraquinones can be achieved in higher yield using 1.1 equiv. of the catalyst PhI(OAc)_2_. Compounds **1**, **3**, **5**, and **7** (1 equiv.) were then used in a substitution reaction to produce aminoanthraquinones derivatives. The reaction was performed with excess BuNH_2_ in the presence of PhI(OAc)_2_ for 15 min to 6 d of reaction, depending on the hindrance of the starting material. BuNH_2_ also acted as a solvent for the reaction (Scheme 2). The catalytic effect was studied by performing the reaction either with or without the catalyst to observe what is the possible reaction or product that might occur or form. Therefore, direct amination of **1** with higher equivalents of butylamine in the presence of 1.1 equiv. of catalyst at RT gave a mixture of anthraquinone butylamines **1a** and **1b** in 1:1 ratio (78% yield). Reducing the equivalents of amine to half either with or without the catalyst produced a single product **1a** in higher yield (70%–90%, Table 2, Entry I, II and III). However, increasing the reaction temperature gave a mixture of products again, where **1a** was isolated as the major one (70%). The butylamino group was observed to substitute at the *ortho* position or replace the OH group. The results proved that the amination occurred *via* a nucleophilic substitution reaction and in agreement with reported works [21,25]. The ^1^H- and ^13^C-NMR spectra of **1a** and **1b** are also consistent with their structures and the reported data.

**Table 2:** Effect of amine equivalence, catalyst and temperature on the amination of **1**, **3**, **5** and** 7**.

Entry	BuNH_2_ (equiv.)	PhI(OAc)_2_ (equiv.)	Temperature	Product (% Yield)
I	450	1.1	RT	**1a **(40), **1b** (38)
II	225	1.1	RT	**1a **(90)
III	225	-	RT	**1a **(70)
IV	225	-	80 °C	**1a **(70), **1b **(15)
V	225	1.1	RT	**3a **(60)
VI	225	-	RT	**3a **(51)
VII	112	1.1	RT	**3a **(46)
VIII	225	1.1	RT	**5a **(7), **5b **(10), **5** (78)
IX	225	1.1	80 °C	**5c **(10), **5d **(73)
X	225	1.1	RT	**1a **(83)
XI	225	-	RT	**1a **(55)
XII	225	-	80 °C	**1a **(46)

**Scheme 3:**
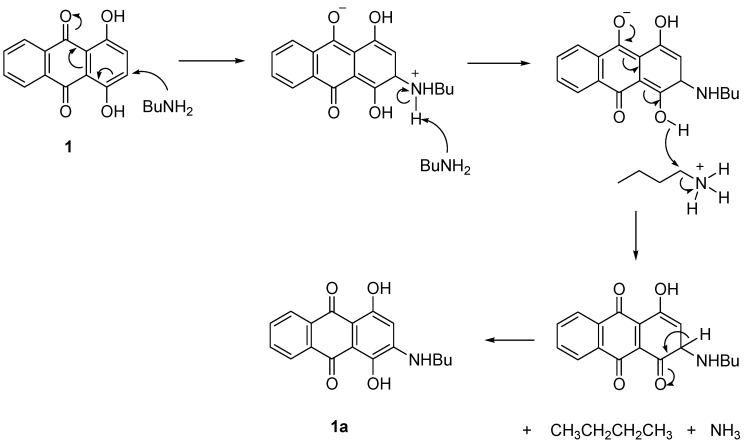
Proposed mechanism of formation of **1a **without using a catalyst.

In the presence of PhI(OAc)_2_, the reaction proceeded with the interaction of the OH group of compound **1** with the catalyst to give the intermediate, O-IPh(OAc). Further attack by BuNH_2_ at the next carbon was produced C=O and removed the PhIOAc. Proton elimination then restored the double bond and OH of **1a** (Scheme 2). In the absence of catalyst, the electron-donating nature of the OH group together with the electron delocalization made compound **1** easily attacked by the BuNH_2_ at the *ortho*-position to form **1a** (Scheme 3).

The compounds **1a**, **1b** and the optimised amount of BuNH_2_ (225 equiv.) and PhI(OAc)_2_ (1.1 equiv.) were used as a comparison of nucleophilic substitution for amination of compounds **3**, **5** and **7**. Therefore, the effect of different 1,4-disubstituted of anthraquinones and the mechanisms were further investigated.

#### 2.2.1. Amination of **3**

**Scheme 4:**
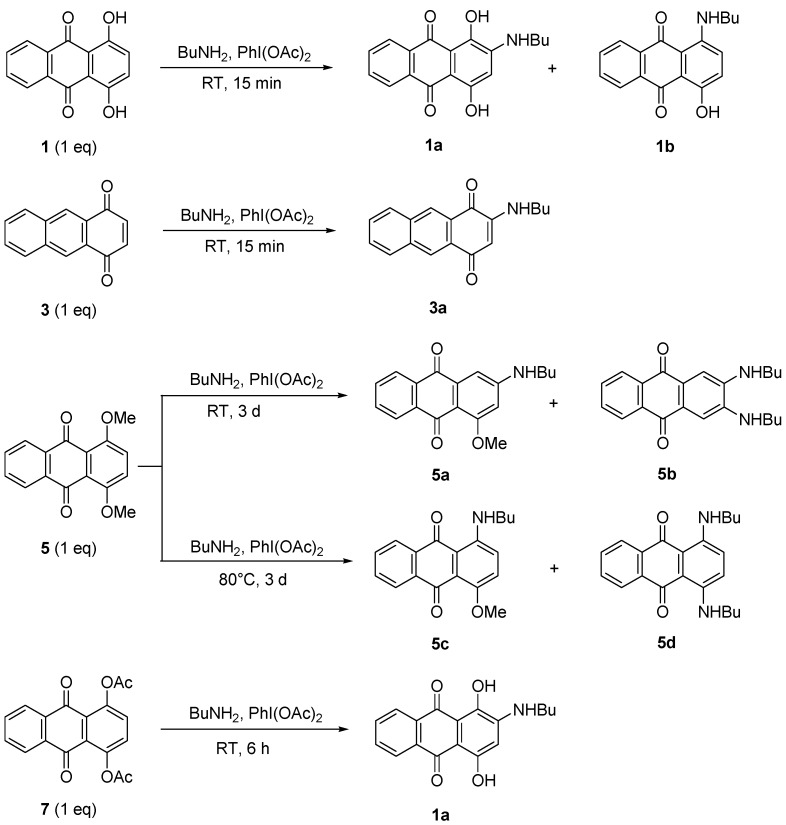
Amination of **1**, **3**, **5**, and **7**.

Amination of compound **3** produced a higher yield of 2-(butylamino)anthracene-1,4-dione (**3a**) when treated with 255 equiv. of BuNH_2_ (Scheme 4). It was observed that the yield decreased when the reaction was performed without a catalyst or if equivalents of amine were reduced (Table 2, Entry V, VI and VII).

**Figure 1:**
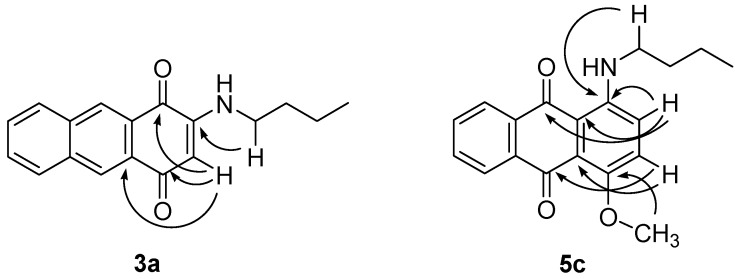
Selected HMBC correlation of **3a** and **5c**.

The ^1^H-NMR spectrum of **3a** showed aromatic proton singlets at *δ* 8.50 and *δ* 8.48 ppm that are attributed to the aromatic carbons at positions C9 and C10, respectively. A proton singlet observed at *δ* 5.80 ppm indicated the presence of an unsaturated proton, whereas a broad singlet observed at *δ* 5.99 ppm referred to the chelated proton of the amine. The ^13^C-NMR spectrum showed the presence of 18 signals correspond to the 18C in the structure. Higher chemical shifts were observed at *δ* 182.7 and *δ* 181.4 ppm that represent the two *C*=O groups, whereas lower signals for C2 and C3 were observed at *δ* 130.2 and *δ* 134.0 ppm. In comparison to the starting material, only one signal was observed for both *C*=O (*δ*184.7), and for C2 and C3 (*δ*140.1) due to the symmetrical structure of **3**. The position of the butylamino group of **3a** was confirmed by an HMBC experiment which showed a *^3^J* correlation as indicated in Figure 1.

**Scheme 5:**
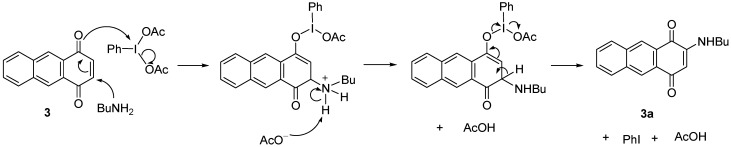
Proposed mechanism of formation of **3a** by using a catalyst.

**Scheme 6:**
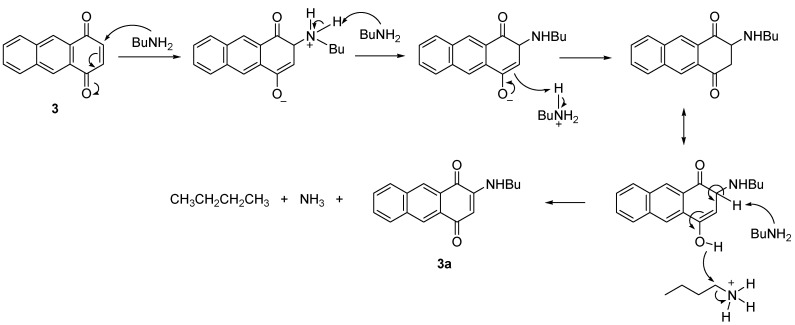
Proposed mechanism for the formation of **3a** (without catalyst).

The proposed mechanism showed that C3 is positively charged and readily attacked by BuNH_2_ due to the resonance that drives by the interaction between the oxygen at C1 with the iodine of the catalyst. Further elimination of a proton at position C3, PhI and AcOH then restored the double bonds between C-C and C-O to give the product **3a **(Scheme 5). A similar resonance effect is assumed to drive the BuNH_2_ to attack the C3 to produce a negatively charged oxygen for the reaction without catalyst. The reaction proceeded through a keto-enol tautomerism followed by elimination of H to give **3a** (Scheme 6). 

#### 2.2.2. Amination of **5**

Treatment of dimethoxyanthraquinone **5** with BuNH_2_ in the presence of PhI(OAc)_2_ produced four aminoathraquinones. At RT, a mixture of 2-(butylamino)-4-methoxyanthracene-9,10-dione (**5a**) and 2,3-(dibutylamino)anthracene-9,10-dione (**5b)** was obtained in low yields (17%) together with the unreacted starting material (78%). A mixture of 1-(butylamino)-4-methoxyanthacene-9,10-dione (**5c**) and 1,4-(dibutylamino)anthracene-9,10-dione (**5d**) was produced at 80 °C, in higher combined yield (83%, Scheme 2). The diaminosubstituted products **5b** and **5d** were isolated as a major product (Table 2, Entry VIII and IX). It appears that the higher the temperature, the more the nucleophilic substitution selectivity. All four amino derivatives were easily separated by column chromatography (dichloromethane-petroleum ether/3:1). It seems that compound **5a** and **5c** which had same *R_f_* values and appearance (*R_f_* 0.33, dichloromethane-petroleum ether, 4:1; dark pink solid) were produced first as intermediates and then reacted further to form diaminoanthraquinones **5b** and **5d**. Surprisingly, compounds **5b** and **5d** also shared same *R_f_* values 0.67 (dichloromethane-petroleum ether, 4:1) and both were obtained as dark blue solids.

The ^1^H-NMR spectra of **5a** and **5c** displayed signals attributed to methoxy protons at *δ* 3.98 and *δ* 3.98 ppm, respectively. A broad singlet signal at *δ* 7.27 ppm in the spectrum of **5a** was assigned to the non-chelated amine proton, whereas for **5c**, the spectrum show a higher chemical shift at *δ* 9.84 ppm which is due to the hydrogen bond between the amine hydrogen and carbonyl oxygen. Both compounds shared the same molecular formulae, C_19_H_19_NO_3_, as the mass spectra exhibited a molecular ion peak at *m/z* 309. Selected HMBC correlations were shown in Figure 1.

The ^1^H-NMR of **5d** showed the disappearance of the signal for the methoxy protons, which clearly indicated that nucleophilic aromatic substitution had occurred. The broad singlet at *δ* 10.73 ppm was assigned to the chelated amine proton. The ^13^C-NMR showed the presence of 11 signals attributed to 22 carbons which are symmetrical. Four quaternary carbons were observed at *δ* 182.0 (*C*=O), 146.2 (*C*-C=O), 134.6 (O=C-*C*) and 109.5 ppm (*C*-NH). Three peaks at *δ* 123.5, 126.0 and 131.8 ppm were assigned to methine aromatic carbons.

**Scheme 7:**
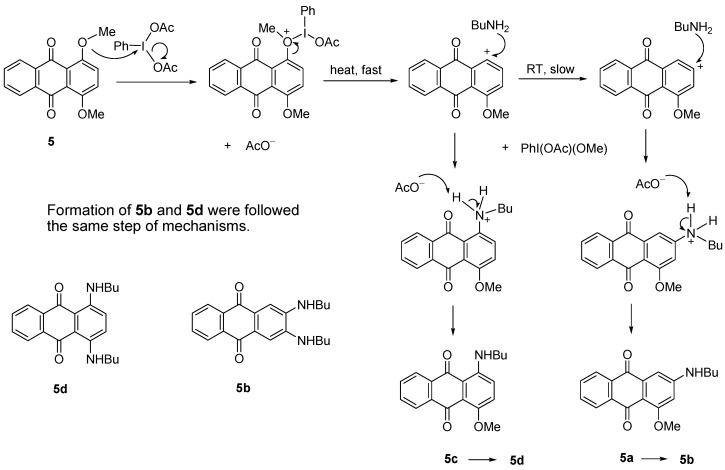
Proposed mechanism of formation of **5a**, **5b**, **5c** and **5d**.

The reaction occurred as the methoxy group interacts with the catalyst. The positive charge on oxygen is then stabilized by the C1-O bond cleavage driven by the formation of PhI(OAc)(OCH_3_) and leaves the aromatic carbocation. With increasing heat, the carbocation is readily attacked by BuNH_2_ to produce **5c** and **5d**. In contrast a slow reaction at RT favored the carbocation rearrangement to produce **5a** and **5b** (Scheme 7). This aromatic substitution of methoxy groups by amines is also supported by literature precedents [21,25,28].

#### 2.2.3. Amination of **7**

**Scheme 8:**
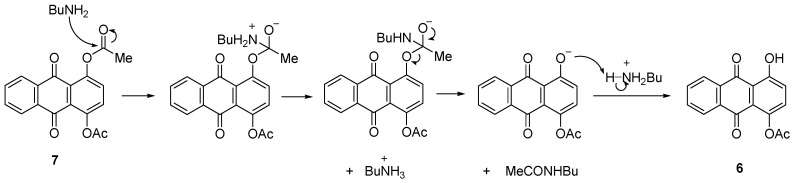
Reduction of acyl group of **7** by butylamine.

2-(Butylamino)-1,4-dihydroxyanthracene-9,10-dione (**1a**) was successfully obtained in 83% yield under a similar amination approach applied to the acylated anthraquinone **7** (Scheme 4). The mechanism suggested that the acetate groups were reduced by the conditions and reagent used to give 1,4-dihydroxyanthraquinone (**1**) as an intermediate before it further reacted with BuNH_2_/PhI(OAc)_2_ to give **1a** (Scheme 8). It was observed that **1a** can still be obtained when the reaction was performed without catalyst or at 80 °C but gave lower yield. Heat applied to the reaction induced the reaction to be faster as the reaction time reduced from 6 h to 4 h (Table 2, Entry X, XI and XII).

The ^1^H-NMR for **1a** showed the disappearance of the COOCH_3_ signal which proved that the acyl groups were reduced. This is also proven by the existence of strong chemical shifts at *δ* 14.23 and *δ* 13.90 ppm which represented the two chelated OH. A broad singlet proton at *δ* 5.60 ppm was attributed to the amine proton. 

All three new aminoanthraquinones **3a**, **5a** and **5b** were subjected to cytotoxic activity assays against MCF-7 (estrogen receptor positive human breast) and Hep-G2 (human hepatocellular liver carcinoma) cancer cell lines with compounds **3a** and **5a** being the most cytotoxic (IC_50_ 1.1–3.0 g/mL). Increasing the number of amino substituents (compound **5b**) on the structure of anthraquinone basically reduced the activity against both MCF-7 and Hep-G2 cancer cell lines with IC_50_ of 3.0 and 13.0 g/mL, respectively (Table 3). Saha *et al.* [34] reported that the starting material, quinizarin **1** (free hydroxy groups) was not active towards MCF-7 but the methylated hydroxyl derivative of quinizarin showed some potential with IC_50_ values 70–80 M. The results of this study also supported their finding where the presence of methoxy or amino groups enhanced the cytotoxic activity of the compounds. 

**Table 3:** Cytotoxic activity of aminoanthraquinones.

Sample	Cytotoxic Activity IC_50_ (µg/mL)	
	MCF-7	Hep-G2
**3a**	1.1	1.2
**5a**	1.1	3.0
**5b**	3.0	13.0

Compounds **3a**, **5c** and **5d** have been screened for antimicrobial activity using four different types of microbe, methicillin-resistant* Staphylococcus aureus* (MRSA), *Pseudomonas aeruginosa*, *Candida albicans* and *Escherichia coli*. The other two new compounds, **5a** and **5b** were replaced by compounds **5c** and **5d** due to insufficient materials and based on the similarity of their structures. All four compounds have the same methoxy and amino substituents and only differ at their amino positions. Therefore, it was assumed that all four compounds might give similar activity results. However, none of compounds had any effect on the growth of all four bacteria or the fungus.

## 3. Experimental

### 3.1. General

All chemicals are commercially available and were of analytical grade and used without purification unless otherwise stated. All reactions were monitored by TLC, using Silica gel 60 F245 (Merck KGaA) precoated aluminium backed plates and visualised by UV and H_2_SO_4_ solution. All organic extracts were dried over Na_2_SO_4_ and evaporated using rotary evaporator. Column chromatography was performed on silica gel 60. Melting points were recorded by digital melting point equipment (Electrothermal IA9000 Series). The IR spectra were obtained by using Perkin-Elmer FT-IR Model Spectrum 100 series spectrophotometer using UATR techniques and the adsorption bands were measured in the range of 280–4000 cm^−1^. MS spectra were recorded using equipped Shimadzu model QP5050A series. The 1D NMR and 2D NMR spectra were run on a JEOL machine at 400 MHz or 500 MHz. All chemical shifts, *δ* were recorded in ppm relative to TMS signal. The coupling constants *J* are given in Hz.

### 3.2. Reduction

1,4-Dihydroxyanthraquinone (**1**, 1 equiv.) was dissolved in methanol (3 mL) and stirred homogenously under a nitrogen atmosphere. Sodium borohydride (1, 3 or 15 equiv.) was added slowly to the reaction mixture and stirred for 30 min. Water (0.5 mL) was added to the reaction mixture and the pH was adjusted to 5–6 with 1 M HCl. Methanol was evaporated and the residue of aqueous layer was extracted with CH_2_Cl_2_ (3 × 10 mL). The combined organic layer were dried, filtered and evaporated under vacuum. The crude product was purified by column chromatography (CH_2_Cl_2_-petroleum ether, 3:2) to obtain compounds **2** (NaBH_4_: 1 equiv.) and **3** (NaBH_4_: 1, 3 and 15 equiv.).

*4-Hydroxyanthracene-1,10-dione* (**2**). Dark orange powder (65.0 mg, 69%); mp 205.8–206.1 °C; v_max_ (UATR) 3317, 3056, 1662, 1568, 1271 cm^−1^; δ_H_ (500 MHz, CDCl_3_) 13.74 (1H, s, OH), 8.44 (1H, d, *J* = 8.0, CH=C), 8.03 (1H, s, CH=C), 7.91 (1H, d, *J* = 8.0, CH=C), 7.64–7.69 (2H, m, H-aromatic), 7.00 (1H, d, *J* = 10.3, H-aromatic), 6.96 (1H, d, J 10.3, H-aromatic); δ_C_ (125 MHz, CDCl_3_) 189.2, 184.1, 162.6, 140.8, 140.0, 136.0, 131.4, 130.6, 129.3, 127.7, 127.4, 124.9, 121.9, 108.9; m/z (EI) 224 (M^+^, C_14_H_8_O_3_ requires 224).

*Anthracene-1,4-dione* (**3**). Orange powder (78.4 mg, 90%); mp 216.4–217.2 °C; v_max_ (UATR) 3319, 3053, 1662, 1596 cm^−1^; δ_H_ (400 MHz, CDCl_3_) 8.55 (2H, s, CH=C), 8.01 (2H, dd, *J* = 2.8, 6.4, H-aromatic), 7.66 (2H, dd, *J* = 2.8, 6.4, H-aromatic), 7.03 (2H, s, H-aromatic); δ_C_ (100 MHz, CDCl_3_) 184.7, 140.1, 134.9, 130.3, 129.7, 128.9, 128.4; m/z (EI) 208 (M^+^, C_14_H_8_O_3_ requires 208).

### 3.3. Methylation

A mixture of **1 **(1 equiv.), K_2_CO_3_ (11 equiv.) and (CH_3_)_2_SO_4_ (14 equiv.) in acetone (5 mL) was refluxed for 3 h. The reaction mixture was added to 25 mL of 2 M HCl and extracted with EtOAc (3 × 25 mL). The combined organic layers were dried, filtered and evaporated under vacuum. The crude product was purified by column chromatography (CH_2_Cl_2_-petroleum ether, 4:1) to produce compounds **4** and **5**.

*1-Hydroxy-4-methoxyanthracene-9,10-dione* (**4**). Orange powder (5.3 mg, 5%); mp 162–163 °C; vmax (UATR) 3073, 2924, 1667, 1590, 1440, 1234, 1178 cm^−1^; δH (400 MHz, CDCl3) 12.71 (1H, s, OH), 8.01 (2H, t, *J* = 6.4, H-aromatic) 7.52 (2H, dt, *J* = 19.3, 6.4, H-aromatic), 7.15 (1H, d, *J* = 9.2, H-aromatic), 7.06 (1H, d, *J* = 10.1, H-aromatic), 3.76 (3H, s, OCH3); δC 188.8, 181.5, 157.2, 154.1, 134.9, 134.6, 133.1, 132.1, 127.2, 126.2, 126.2, 123.5, 119.0, 115.8, 56.9 (100 MHz, CDCl3); m/z (EI) 254 (M+, C15H10O4 requires 254).

*1,4-Dimethoxyanthracene-9,10-dione* (**5**). Yellow powder (108.1 mg, 96%); mp 198–200 °C; v_max_ (UATR) 3089, 2998, 1668, 1566, 1404, 1243 cm^−1^; δ_H_ (400 MHz, CDCl_3_) 8.10 (2H, dd, *J* = 5.5, 3.6 H-aromatic), 7.65 (2H, dd, *J* = 5.5, 3.6 H-aromatic), 7.28 (2H, s, H-aromatic), 3.94 (6H, s, OCH_3_); δ_C_ (100 MHz, CDCl_3_) 183.5, 154.2, 134.3, 133.4, 126.5, 123.1, 120.3, 57.1; m/z (EI) 268 (M^+^, C_16_H_12_O_4_ requires 268).

### 3.4. Acylation

A mixture of compound **1 **(1 equiv.), Ac_2_O (33 equiv.), and NaOAc (1 equiv) was stirred at 100 °C for 2 h. The reaction mixture were added to ice cold water and extracted with CH_2_Cl_2_ (3 × 25 mL). The combined organic layers was dried and evaporated under vacuum. The crude product was purified by column chromatography (CH_2_Cl_2_-petroleum ether, 3:1) to produce compounds **6** and **7**.

*4-Hydroxy-9,10-dioxo-9,10-dihydroanthracen-1-yl acetate* (**6**). Light orange powder (31.8 mg, 26%); mp 189.1–189.5 °C; v_max_ (UATR) 3535, 2935, 1772, 1667, 1587, 1360, 1168, 1011 cm^−1^; δ_H_ (400 MHz, CDCl_3_) 13.04 (1H, s, OH), 8.28 (1H, t, *J* = 4.6, H-aromatic), 8.21 (1H, t, *J* = 4.6, H-aromatic), 7.79 (2H, t, *J* = 3.7, H-aromatic), 7.33 (2H, s, H-aromatic), 2.46 (3H, s, OCH_3_ ); δ_C_ (100 MHz, CDCl_3_) 188.5, 181.2, 169.9, 161.3, 143.2, 135.0, 134.1, 133.5, 132.5, 131.22, 127.4, 126.8, 126.0, 123.5, 115.9, 21.2; m/z (EI) 282 (M^+^, C_16_H_10_O_5_ requires 282).

*9,10-Dioxo-9,10-dihydroanthracene-1,4-diyl diacetate* (**7**). Light yellow powder (122.4 mg, 90%); mp 236.2–237.0 °C; v_max_ (UATR) 3081, 1762, 1667, 1582, 1437, 1177 cm^−1^; δ_H_ (400 MHz, CDCl_3_) 8.14 (2H, dd, *J* = 5.5, 3.7 H-aromatic), 7.73 (2H, dd, *J* = 5.5, 3.7, H-aromatic), 7.41 (1H, s, H-aromatic), 2.47 (6H, s, OCCH_3_ ); δ_C_ (100 MHz, CDCl_3_) 181.6, 169.6, 148.3, 134.2, 133.4, 131.1, 127.0, 126.2, 21.3; m/z (EI) 324 (M^+^, C_16_H_10_O_5_ requires 324).

### 3.5. Amination—General Procedure

Butylamine (112, 225 or 450 equiv.) was added dropwise to a mixture of **1**, **3**, **5**, or **7** (1 equiv.) and PhI(OAc)_2_ (0.41 mmol, or without catalyst) at RT (or heated at 80 °C) and stirred for 15 min to 3 d. The reaction mixture, 25 mL 10 M HCl and NaHCO_3_ (84 mL) were added to ice cold water (84 mL) successively. The resulting solution was extracted with EtOAc (3 × 25 mL). The organic layer was washed with H_2_O (3 × 25 mL), dried and evaporated. The crude product was then chromatographed on silica gel (DCM-petroleum ether, 4:1) to give a mixture of amine derivatives of anthraquinone **1a**, **1b**, **3a**, **5a**–**d**.

*2-(Butylamino)-1,4-dihydroxyanthracene-9,10-dione* (**1a**). Dark pink powder (176.0 mg, 90%); mp 158.0–158.4 °C; v_max_ (UATR) 3378, 3308, 2949, 1635, 1565, 1516, 1459, 1417, 1260, 1156 cm^−1^; δ_H_ (500 MHz, CDCl_3_) 14.23 (1H, s, OH) 13.90 (1H, s, OH), 8.28 (1H, d, *J* = 6.9, H-aromatic), 8.25 (1H, d, *J* = 6.9, H-aromatic), 7.75 (1H, t, *J* = 5.8, H-aromatic), 7.68 (1H, t, *J* = 5.8, H-aromatic), 6.05 (1H, s, H-aromatic), 5.57 (1H, br. s, NH), 3.21 (2H, q, *J* = 6.9, NHCH_2_CH_2_), 1.65–1.72 (2H, m, CH_2_CH_2_CH_2_), 1.42–1.50 (2H, m, CH_2_CH_2_CH_3_), 0.98 (3H, t, J 6.9, CH_2_CH_3_); δ_C_ (125 MHz, CDCl_3_) 183.5, 178.0, 166.5, 153.6, 147.7, 134.7, 134.0, 132.4, 132.3, 126.5, 126.3, 110.4, 102.9, 100.3, 42.8, 30.7, 20.3, 13.7; m/z (EI) 311 (M^+^, C_18_H_17_NO_4_ requires 311). 

*1-(Butylamino)-4-hydroxyanthracene-9,10-dione* (**1b**). Dark purple powder (31.5 mg, 38%); mp 127.2–127.9 °C; v_max_ (UATR) 3170, 2954, 2926, 2853, 1616, 1580, 1463, 1233, 1162 cm^−1^; δ_H_ (500 MHz, CDCl_3_) 13.71 (1H, s, OH) 10.29 (1H, br. s, NH), 8.30 (2H, dd, *J* = 8.0, 16.0, H-aromatic), 7.77 (1H, t, *J* = 5.7, H-aromatic), 7.70 (1H, t, *J* = 5.8, H-aromatic), 7.21 (1H, s, H-aromatic), 7.20 (1H, s, H-aromatic), 3.36 (2H, t, *J* = 6.9, NHCH_2_CH_2_), 1.70–1.77 (2H, m, CH_2_CH_2_CH_2_), 1.47–1.55 (2H, m, CH_2_CH_2_CH_3_), 0.99 (3H, t, *J* = 6.9, CH_2_CH_3_); δ_C_ (125 MHz, CDCl_3_) 187.5, 182.0, 156.8, 147.7, 135.5, 134.2, 132.7, 132.5, 129.0, 126.7, 126.4, 124.1, 113.8, 108.4, 42.7, 31.6, 20.4, 13.9; m/z (EI) 311 (M^+^, C_18_H_17_NO_3_ requires 295).

*2-(Butylamino)anthracene-1,4-dione* (**3a**). Yellow powder, (70.1 mg, 60%); mp 173.4–173.9 °C; v_max_ (UATR) 3325, 2926, 1677, 1573, 1504, 1456, 1406, 1319, 1250 cm^−1^; δ_H_ (500 MHz, CDCl_3_) 8.50 (1H, s, H-aromatic), 8.48 (1H, s, H-aromatic), 7.93 (2H, t, *J* = 8.0, H-aromatic), 7.59 (1H, t, *J* = 5.8, H-aromatic), 7.55 (1H, d, *J* = 8.1, H-aromatic), 5.99 (1H, br. s, NH), 5.80 (1H, s, CH=C), 3.16 (2H, q, *J* = 6.9, NHCH_2_CH_2_), 1.62–1.69 (2H, m, CH_2_CH_2_CH_2_), 1.37–1.46 (2H, m, CH_2_CH_2_CH_3_), 0.94 (3H, t, *J* = 6.9, CH_2_CH_3_); δ_C_ (125 MHz, CDCl_3_) 182.7, 181.4, 149.1, 135.7, 134.0, 130.2, 130.0, 129.8, 129.6, 129.0, 128.7, 127.7, 127.6, 102.6, 42.4, 30.4, 20.3, 13.8; m/z (EI) 279 (M^+^, C_18_H_17_NO_2_ requires 279).

*2-(Butylamino)-4-methoxyanthracene-9,10-dione* (**5a**). Dark purple powder (12.5 mg, 10%); mp 87.8–88.2 °C; v_max_ (UATR) 3535, 2936, 1630, 1590, 1506, 1461, 1354, 1250, 1180 cm^−1^; δ_H_ (400 MHz, CDCl_3_) ) 8.23 (1H, t, *J* = 2.8, H-aromatic), 8.21 (1H, t, *J* = 2.8, H-aromatic), 7.70 (2H, t, *J* = 3.6, H-aromatic), 7.37 (1H, d, *J* = 9.2, H-aromatic), 7.27 (1H, d, *J* = 10.1, H-aromatic), 5.29 (1H, br. s, NH), 3.98 (3H, s, OCH_3_), 3.33 (2H, t, *J* = 7.3, NHCH_2_CH_2_), 1.73–1.82 (2H, m, CH_2_CH_2_CH_2_), 1.46–1.57 (2H, m, CH_2_CH_2_CH_3_), 0.99 (3H, t, *J* = 7.3, CH_2_CH_3_); δ_C_ (100 MHz, CDCl_3_) 185.3, 183.3, 152.7, 145.6, 134.5, 134.0, 133.3, 133.2, 126.8, 126.2, 124.1, 121.7, 121.4, 114.0, 57.6, 44.1, 31.1, 20.4, 13.9; m/z (EI) 309 (M^+^, C_19_H_19_NO_3_ requires 309).

*2,3-(Dibutylamino)anthracene-9,10-dione* (**5b**). Dark blue powder (10.6 mg, 7%); mp 113.0–113.6 °C; v_max_ (UATR) 3539, 2929, 1669, 1583, 1522, 1364, 1249, 1172 cm^−1^; δ_H_ (500 MHz, CDCl_3_) 8.29 (2H, dd, *J* = 5.7, 3.4, H-aromatic), 7.76 (2H, d, *J* = 5.7, 3.4, H-aromatic), 7.50 (2H, s, H-aromatic), 5.29 (2H, s, NH), 3.36 (4H, t, *J* = 6.9, NHCH_2_CH_2_), 1.79–1.85 (4H, m, CH_2_CH_2_CH_2_), 1.47–1.54 (4H, m, CH_2_CH_2_CH_3_), 0.99 (6H, t, *J* = 6.9, CH_2_CH_3_); δ_C_ (500 MHz, CDCl_3_) 184.5, 141.6, 133.8, 133.7, 126.8, 125.4, 114.7 , 45.9, 30.6, 20.3, 13.8; m/z (EI) 350 (M^+^, C_22_H_26_N_2_O_2_ requires 350).

*1-(Butylamino)-4-methoxyanthacene-9,10-dione* (**5c**). Dark purple powder (11.4 mg, 10%); mp 96.3–96.9 °C; v_max_ (UATR) 3536, 2929, 1647, 1591, 1506, 1461, 1356, 1248, 1180 cm^−1^; δ_H_ (500 MHz, CDCl_3_) 9.84 (1H, s, NH), 8.17 (2H, dd, *J* = 16.9, 2.3, H-aromatic), 7.60–7.67 (2H, m, H-aromatic), 7.27 (1H, d, *J* = 9.2, H-aromatic), 7.02 (1H, d, *J* = 9.2, H-aromatic), 3.92. (3H, s, OCH_3_), 3.23 (2H, q, *J* = 6.9, NHCH_2_CH_2_), 1.66–1.71 (2H, m, CH_2_CH_2_CH_2_), 1.43–1.51 (2H, m, CH_2_CH_2_CH_3_), 0.96 (3H, t, *J* = 6.9, CH_2_CH_3_); δ_C_ (125 MHz, CDCl_3_) 184.8, 183.4, 151.6, 147.5, 134.4, 134.2, 133.0, 132.8, 126.6, 126.1, 124.3, 121.1, 120.2, 112.3, 57.5, 42.8, 31.4, 20.5, 13.9; m/z (EI) 309 (M^+^, C_19_H_19_NO_3_ requires 309).

*1,4-(Dibutylamino)anthracene-9,10-dione* (**5d**). Dark blue powder (94.5 mg, 73%); mp 113.5–114.2 °C; v_max_ (UATR) 3747, 2927, 1641, 1570, 1518, 1461, 1364, 1258 cm^−1^; δ_H_ (500 MHz, CDCl_3_) 10.73 (2H, br. s, NH), 8.28 (2H, dd, J 5.7, 3.4, H-aromatic), 7.62 (2H, dd, *J* = 5.7, 3.4, H-aromatic), 7.04 (2H, s, H-aromatic), 3.25–3.29 (4H, m, NHCH_2_CH_2_), 1.65–1.71 (4H, m, CH_2_CH_2_CH_2_), 1.43–1.51 (4H, m, CH_2_CH_2_CH_3_), 0.96 (6H, t, *J* = 6.9, CH_2_CH_3_); δ_C_ (125 MHz, CDCl_3_) 182.0, 146.2, 134.6, 131.8, 126.0, 123.5, 109.5 , 42.6, 31.8, 20.5, 14.0; m/z (EI) 350 (M^+^, C_22_H_26_N_2_O_2_ requires 350).

### 3.6. Cytotoxic Assays

The cytotoxic assay was carried out according to the method described by Sukari *et al.* [35]. The MCF-7 (estrogen receptor positive human breast) and Hep-G2 (human hepatocellular liver carcinoma) cancer cells were purchased from ATCC. The cells were grown and maintained in RPMI 1640 media, supplemented with 10% fetal calf serum (FCS) and 1% antibiotic penicillin-streptomycin in an atmosphere of 5% CO_2_ at 37 °C. The medium was used to dilute the cells to a concentration of 5 × 10^5^ cells/mL. From this cells suspension, 100 μL of various concentrations of the synthesised compounds were pipetted into a 96-well micro titer plate and incubated in 37 °C, 5% CO_2_ incubator for 72 h. The various concentration used were 100, 50, 25, 12.5, 6.25, 3.125, 1.56 μg/mL. The assay of each concentration of synthesised compounds was performed in triplicate and the control wells of untreated population were also included. After three d, the fraction of surviving cells was determined relative to the untreated cells population by the colorimetric MTT (3-(4,5-dimethylthiazol-2-yl)-2,5-diphenyltetrazolium bromide) method where the viability of cells was measured by 20 μL of blue formazan crystals of MTT solution (5 mg/mL in phosphate-buffered saline, PBS) added to each well followed by incubation in 37 °C, 5% CO_2_ incubator for 3–4 h. 100 μL of cells suspension or cells monolayer in each micro titer was removed from each well. The plate was left at room temperature for 30 min before reading the absorbance. The absorbance was read with the multiwell scanning spectrophotometer (ELISA reader) test wavelength of 570 nm and reference wavelength of 630 nm. The cytotoxic index used was IC_50_ which is the concentration that yield 50% inhibition of the cells compared with untreated control.

### 3.7. Antimicrobial Assay

The modified disc diffusion method was followed a procedure by Garba and Okeniyi [36]. Nutrient agar (20 g) was suspended in 1 L of distilled water and stirred. It was boiled to dissolve homogenously and autoclaved at 121 °C for 20 min. The agar was allowed to cool to 50 °C and poured into sterile disposable petri dishes. Four microbes, methicillin-resistant *Staphylococcus aureaus* (MRSA), *Pseudomonas aeruginosa*, *Candida albicans* and *Escherichia coli* were inoculated into prepared nutrient broth and incubated at 37 °C for overnight. The suspension of the microbes in the broth was inoculated on the nutrient agar using sterile cotton bud. The sterile 6 mm paper discs were impregnated with the synthesised compounds in concentrations of 20, 10, 5, 2, 1, 0.5 and 0.1 mg/mL and allowed to soak for 1 min. The paper discs were removed, dried and placed on the surface of agar plates inoculated with the microbial cultures. Each synthesised compound was tested in triplicate. Paper discs impregnated with acetone were used as a negative control. The petri dishes were incubated in inverted position at 37 °C for 24 h. The zones of inhibitions (clear area without bacterial growth) were measured in cm. 

## 4. Conclusions

Aminoantraquinone derivatives were synthesised through methylation, reduction or acylation then followed by amination. In total, seven aminoanthraquinones (compounds **1a**, **1b**, **3a**, and **5**–**d**) were produced, including three new compounds: 2-(butylamino)anthracene-1,4-dione (**3a**), 2-(butyl- amino)anthracene-9,10-dione (**5a**) and 2,3-(dibutylamino)anthracene-9,10-dione (**5b**). All amino-anthraquinones were produced *via* nucleophilic substitution mechanisms. Aminoanthraquinones **3a**, **5a** and **5b** were found to demonstrate strong cytotoxic activity against both MCF-7 (IC_50_ 1.1, 1.1 and 3.0 µg/mL respectively) and Hep-G2 cancer cell lines (IC_50_ 1.2, 3.0 and 13.0 µg/mL, respectively).
